# Website Redesign of a 16-Week Exercise Intervention for People With Spinal Cord Injury by Using Participatory Action Research

**DOI:** 10.2196/13441

**Published:** 2019-12-17

**Authors:** Maria Cole, Katherine Froehlich-Grobe, Simon Driver, Ross Shegog, Jeffery McLaughlin

**Affiliations:** 1 Baylor Scott and White Institute for Rehabilitation Dallas, TX United States; 2 University of Texas Health Science Center Houston School of Public Health Houston, TX United States; 3 Radiant Digital Vienna, VA United States

**Keywords:** internet, exercise, intervention, spinal cord injury, community-based research

## Abstract

**Background:**

People with spinal cord injury (SCI) are at higher risk for numerous preventable chronic conditions. Physical activity is a protective factor that can reduce this risk, yet those with SCI encounter barriers to activity and are significantly less likely to be active. Limited evidence supports approaches to promote increased physical activity for those with SCI.

**Objective:**

Building upon our previous theory- and evidence-based approach to increase participation in regular physical activity for those with SCI, this study aimed to use a participatory action research approach to translate a theory-based intervention to be delivered via the Web to individuals with SCI.

**Methods:**

A total of 10 individuals with SCI were invited to participate in consumer input meetings to provide the research team with iterative feedback on an initial website designed as a platform for delivering a theory-based exercise intervention.

**Results:**

A total of 7 individuals with SCI whose average age was 43.6 years (SD 13.4) and lived an average age of 12.5 years (SD 14.9) with SCI met on 2 occasions to provide their feedback of the website platform, both on the initial design and subsequently on the revamped site. Their iterative feedback resulted in redesigning the website content, format, and functionality as well as delivery of the intervention program.

**Conclusions:**

The substantially redesigned website offers an easier-to-navigate platform for people with SCI with greater functionality that delivers information using a module format with less text, short video segments, and presents more resources. Preliminary testing of the site is the next step.

## Introduction

### Background

Clinical practice guidelines published by the Consortium for Spinal Cord Medicine recognize that people with spinal cord injury (SCI) face greater risk for cardiometabolic disease (CMD) than the general population. CMD refers to the presence of at least 3 of 6 chronic disease risk factors that include abdominal adiposity, dyslipidemia, hypertension, insulin resistance or glucose intolerance, proinflammatory state, and prothrombotic state [1]. The consortium posits that CMD may be more challenging to treat in those with SCI than the general population and advocates for aggressive prevention. Lifestyle intervention is recommended as the first line of treatment to reduce CMD risk, with a focus on nutrition (ie, follow a heart-healthy diet) and physical activity. Furthermore, the consortium strongly recommends that people with SCI do at least 150 min of physical activity each week beginning as soon as possible after acute SCI [2], in line with national physical activity guidelines for all Americans [3].

Nevertheless, evidence is limited regarding effective approaches to promote regular participation in physical activity for people living with chronic SCI. Although several studies have examined barriers to physical activity that people with SCI face, people living with SCI encounter lack of access to timely and quality health information (eg, SCI-related medical issues and regarding fitness or health promotion) [4] and have fewer opportunities to engage in community-based physical activity than those without a disability [5]. Vissers et al [6] and Levins et al [7] reported that people with SCI described both environmental barriers such as inaccessible buildings, lack of available programs, and societal attitudes as well as personal factors that were barriers such as physical and mental health problems and concerns about body image [6]. Transportation continues to be a leading barrier to participation for people with SCI [8,9] across various health-related lifestyle changes, including physical activity. Given pervasive transportation difficulties facing people with SCI, the internet may offer a feasible and promising approach to help bridge the transportation barrier in delivering interventions that promote health for people with SCI.

### The Potential of Internet Use for Intervention

The internet has dramatically transformed how we conduct our daily lives, and according to the Pew Research Center [10], nearly 9 in 10 adults reported accessing the internet in 2018. Nearly three-quarters (73%) of Americans have home broadband access, and 37% of Americans report using smartphones as their primary means of accessing the internet [11,12]. Although people with SCI report lower rates of internet access, 2 studies [13,14] published in the last decade indicate that 65% to 70% people have computer access, and most of these individuals (63%-92%) use the internet.

Thus, the internet offers a potentially promising platform to connect with individuals living with SCI who face transportation barriers and reside in communities with fewer accessible physical activity options. The internet is increasingly used to deliver relatively low-cost health behavior change programs to populations with chronic health problems such as diabetes [15], cancer [16], and asthma [17]. Furthermore, the internet has successfully been used to promote physical activity [18]. Despite increased use among people with SCI [14,19], there has been substantially less use of the internet to connect with people with SCI. Yet, a handful of internet-based studies have been conducted over the last 3 years that have provided participants with SCI 8 to 12 weeks of website access that included physical therapy education [20], taught self-management strategies to increase frequency of intermittent catheterization [21], reduced depressive symptoms [22,23], and reduced pain [22,24]. Another study delivered a single 60 min Web-based program to provide transfer training [25]. The others delivered educational content via the Web and provided participants with weekly phone calls [20,21,23,24] or email [23,26] by a study staff member. Several taught self-management strategies [21-24], and one used audio [22] and 2 used videos [22,24] to help participants visualize the lessons. Although not an internet-based intervention, researchers tested the effectiveness of delivering an 8-week telehealth program where participants received weekly one-on-one video conference calls with a counselor to promote leisure time physical activity that yielded significant increases in self-reported leisure time physical activity [27].

Although the internet remains an underutilized strategy for reaching and delivering health promotion programs for those with SCI, these initial results of technology use for delivering health education to people with SCI are promising [20]. This paper has described the process and outcome of using a participatory action research (PAR) approach to translate a theory-based, telephonic intervention that targeted increased participation in regular physical activity to be delivered in a group-based setting over the internet.

## Methods

### Design

#### Workout on Wheels Telephone Intervention

Our Workout on Wheels internet intervention (WOWii) program translated a theory- and evidence-based 6-month health behavior intervention, Workout on Wheels (WOW), which was originally tested with a sample of wheelchair users, in a randomized controlled trial [28]. The WOW program was delivered using the combined approach of convening a group-based, day-long educational kick-off session followed by one-on-one telephone calls by intervention staff over 12 months. The formal WOW curriculum was delivered over 6 months, with the core curriculum presented at the workshop plus over 4 months of weekly one-on-one phone calls that tapered over months 5 and 6.

WOW trial participants received a binder of written materials to review during the 6-hour educational kick-off workshop. The WOW trial yielded significant between group differences in time spent in aerobic exercise; however, the intervention group achieved only one-third (approximately 55 min) of the recommended 150 min of weekly cardiovascular activity [28]. WOW participants were observed making social connections at the educational workshop, asked when they could meet again as a group, and expressed interest to stay connected with their peers after the intervention. Thus, WOWii is a direct adaptation of the evidence-based self-management WOW program to a platform where it could be delivered in a group-based format over the internet.

#### Modified Workout on Wheels Internet Intervention

The prototype WOWii website comprised material from the WOW program. The site provided a menu with 8 selections: a home page (featuring images of wheel chair users, an indicator of the user’s achievements in completing exercise planning tasks, and group meeting reminder and hot link), an exercise guide (eg, describes the health benefits, addresses exercise barriers exercise, exercise options for individuals with disabilities, safety issues), links to engage in exercise planning tasks (eg, goal setting, identifying exercise barriers and solutions, listing support people, describing reasons to exercise, tracking exercise, solving barriers to exercise, exercise goals, and exercise tracking), link for tracking their exercise, resources (eg, short bios with pictures of wheelchair users and their exercise programs), achievements, a discussion forum, and leaderboard. The WOWii prototype site images are displayed in Multimedia Appendix 1. The website contained substantial text from the written materials distributed in the previous trial that addressed the health benefits and safety considerations of physical activity (accompanied by images of wheelchair users engaged in various sports and physical activities) and steps to develop and complete their individual exercise plan.

WOWii was abridged to a 16-week program that allows adequate time to teach the curriculum and members to get to know one another. The vision of WOWii was to retain the content and approach of explicitly teaching individuals with SCI self-management strategies to start and maintain an exercise program, while harnessing the power of peer support to facilitate conversation that allows individuals the opportunity and venue to share their knowledge, barriers, and successes as they initiate a new health behavior. WOWii includes 2 interactive components: (1) an internet site that allows self-directed learning via electronic modules organized with weekly content and (2) online group meetings to discuss and practice the weekly lessons facilitated by study staff.

PAR offers an approach to invite members of the community of interest to collaborate with the research team, which increases the relevance and ease of use of the research approach, procedures, and outcomes for the target group [29]. We selected to follow a PAR framework to develop a partnership with individuals living with chronic SCI to ensure that the WOWii website and its content, including the language, layout, and functionality, accurately and adequately addressed the interests and needs of people living with SCI. Focus groups and interviews represent 2 of the most common methods used in PAR to generate data. Gathering people with characteristics similar to the study population into one-on-one meetings or small groups of 7 to 12 provides ideal settings for individuals to discuss their perspectives and concerns with researchers. This approach allows researchers and members of the target community to collaborate on addressing a common goal [30].

### Recruitment

Former SCI inpatients from our rehabilitation hospital who provided written consent to be contacted for future studies were emailed or handed a flier inviting them to participate in focus group meetings. Interested individuals contacted a study staff member to learn more about the study and enroll if eligible. Eligibility criteria included individuals between the ages of 18 and 65 years; had SCI for at least six months, that is, at a C6 level or below that requires wheelchair use; and have access to a computer with internet access. Injury level was included to help ensure that potential participants would have the finger function necessary to independently navigate the website. No criteria were established regarding physical activity participation to ensure those who were both active and inactive participated. Participants completed a 4-item physical activity history questionnaire that asked about their activities postinjury: (1) did any moderate or vigorous physical activities that caused an increase in their breathing or heart rate, (2) the number of days per week, (3) the number of minutes per day, and (4) the type of activities performed.

### Consumer Input and Data Collection and Analysis

Input was provided by 7 people with SCI over 2 rounds of individual and group meetings. Our approach for incorporating their input was informed by qualitative approaches to identify common themes. During the first round of input, participants provided feedback on the WOWii site. The research team convened 3 sessions (each with 1 to 4 people) for individuals to share their feedback and offer input about the content and usability of the website’s initial design. Participants were asked to review the website for at least 30 min on their own before the first meeting to allow adequate time to engage in brainstorming activities, generate novel ideas, and provide comments about the existing content.

Facilitators led a semistructured conversation following the Liberating Structures approach to yield generative, open discussions. Liberating Structures [31] emerged from business as an alternative approach to traditional meetings that can include inflexible power dynamics that may discourage impartial feedback. The Liberating Structures framework was first used to permit frontline workers to have more opportunities to work together to develop and offer innovative ideas with their leadership. The facilitators chose this approach to best align with the overall PAR framework centering on the participants’ lived experiences and empowering them to partner with the researchers in the website redesign process. The facilitators used the 1-2-4-ALL design and the 25/10 Crowd Outsourcing approaches during the first half of the initial session to facilitate discussion. The 1-2-4-ALL design asked the group to respond to an open-ended question by spending 1 min alone, 2 min in pairs, 4 min in foursomes, and then 5 in the entire group. This time-limited approach produced several ideas and helped to keep the focus group meeting on schedule. The 25/10 Crowd Outsourcing approach asked participants to envision their boldest response to an open-ended question and write it on an index card. The cards were passed to other group members who then scored their ideas on a scale of 1 (low) to 5 (high). The cards were passed around for 5 scoring rounds, then the facilitator asked which participants were holding cards with a score of 25. The facilitators continued to ask for the highest ranked ideas in decreasing ranked order (eg, scored 24, 23, 22…) until the top 10 ideas were collected. This approach allowed the facilitators to understand what type of website design the participants envisioned and to gain a sense of which ideas were most salient. Participants responded to questions such as “If money wasn’t a limitation, what would you create on this website?” During the last half of the session, participants were asked to identify their primary likes and dislikes and rate the credibility of information and ease of use of the website.

The facilitators took written notes and audio recorded each session for reference purposes to ensure that handwritten notes shared with the larger research team were complete. The team reviewed comments from the recordings and notes, and then grouped the comments into categories based on thematic content that emerged. Comments were broadly organized based on recommendations to add, revise, or delete content. The team discussed all suggested changes and then prioritized the recommendations, giving greater weightage to modifications that were likely to have greater impact on changing behavior, given the time and budgetary constraints that prevented our implementing all recommended changes. The team worked closely with the website developer to execute suggested changes.

A second round of 90-min meetings with the original participants (2 separate meetings to accommodate the 7 individuals’ schedules) were convened to obtain feedback on the new WOWii design, content, and appearance. Participants reviewed the website content while it was projected onto a screen to facilitate group discussion regarding whether the changes achieved their intended purpose and gather additional comments. Unfortunately, owing to time and funding constraints in meeting our timelines, the participants did not receive the website link to explore the revised site before the meeting. Participants provided substantially fewer recommendations during this feedback round. Their comments about the redesigned website concentrated on the flow and functionality of the site and predominantly addressed how to refine the new content.

## Results

### Overview

A total of 7 individuals who lived an average age of 12.5 years (SD 14.9; range 1-42 years) with SCI provided input. All individuals used a manual wheelchair, most experienced paraplegia (n=5), although 2 had tetraplegia. More than half were male (n=4), all were white, and their average age was 43.6 years (SD 13.4; range 26-60 years). Individuals in the sample were well educated, having earned a bachelor’s degree (n=4) or greater (n=3) and more than two-thirds (71%, 5/7) were employed. Participants suggested an array of website enhancements that were characterized as being in 1 of 3 categories: (1) design, (2) content, and (3) functionality and program delivery, presented in Table 1 organized by which round the feedback was provided. These enhancements are described in detail in the following paragraphs.

**Table 1:** Consumer input participants (n=7).

Demographic Characteristics		Values
**Sex, n**		
	Male	4
	Female	3
Age (years), mean (SD)		43.57 (13.40)
Time since injury, mean (SD)		12.50 (14.94)
**Spinal cord injury, n**		
	Quadriplegia	1
	Paraplegia	6
**Race, n**		
	White	7
**Ethnicity, n**		
	Hispanic	1
	Non-Hispanic	6
**Marital status, n**		
	Married	4
	Widowed	1
	Never been married	2
**Education, n**		
	Bachelor’s degree	4
	Master’s degree	2
	Other graduate degree	1
**Employment status, n**		
	Not currently employed	1
	Employed part-time	3
	Employed full-time	2
**Household income (US $), n**		
	20,000-24,999	1
	60,000-69,999	1
	100,000 or more	4
**Report being physically active since injury (n=4),** **mean (SD)**		
	Days spent in moderate or vigorous activity	3.0 (2.3)
	Minutes spent in moderate or vigorous activities^a^	112.50 (102.07)

^a^Activities reported: wheelchair tennis, rugby, hand cycling, swimming, wheeling, and weight training.

### Design Changes

**Figure 1:**
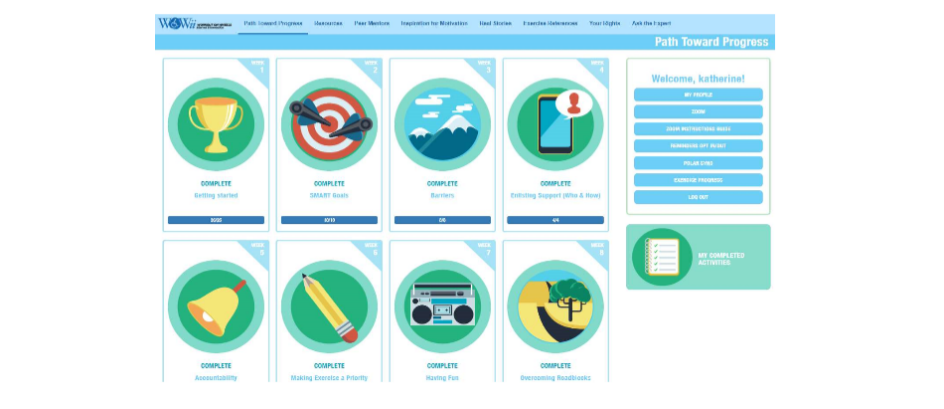
Website screenshot of modules and resource tabs.

The most substantial changes made to the website design addressed participants’ concerns about the organization and flow of site content. Participants strongly encouraged the team to provide users a roadmap by reorganizing the content to clearly indicate the order in which someone should review information. The website was substantially redesigned, with the focus on easy-to-navigate weekly learning modules that covered topics designed to introduce and teach self-management strategies (eg, setting goals, tracking progress, rewarding success, addressing barriers, and solving problems) that would facilitate developing a realistic exercise plan that considered changes they would make to their daily routine. Each module introduced the topic, provided examples, and included a theory-based skill building activity for participants to practice. The original WOWii included the same self-management topics, and the redesign targeted the format for presenting the content within the modules (see Table 2 and Figure 1). Topics covered in each module (module topics listed in Table 3) will be the topic discussed in that week’s virtual session, which will be delivered using a commercially available communication platform that allows videoconferencing such as Skype, Zoom, or WebEx. The participants also discussed allowing individual users to interact with each other on the site. Although budget and time constraints prevented adding this feature, the team recognized that an array of options outside the site do allow for this type of interaction. Thus, the study team opted to create a private Facebook group that participants could join and would be moderated by a WOWii researcher.

**Table 2:** Website changes recommended during each round of participant meetings.

Categories	Round 1			Round 2	
	Implemented	Not implemented	Implemented		Not implemented
Design	Add roadmap for users Present content using e-modules Enlarge font Add option to customize profile photo	Allow user interaction	—^a^		—
Content	Reduce volume of text Add videos Provide additional resources Provide YouTube links	Add calendar of events Provide accountability partners	Add motivational statements Add peer intro videos Created a downloadable pdf of resources Delete RPE^b^ scale as intensity indicator Add more graphics		—
Functionality and program delivery	Add federal legislation information Include peer mentors in delivery Add 'Ask the Expert' link Offer text reminders for exercise Display weekly PA^c^ goals in relation to PA achieved Optimize site for mobile access	Assess stage of change Add calorie burn guide	—		—
Edits	—	—	Make wording changes in modules Recommend formatting changes Simplify language		—

^a^Not applicable.

^b^RPE: Rating of Perceived Exertion Scale.

^c^PA: physical activity.

**Table 3:** List of module and virtual session topics.

Month and week		Topic
**Month 1**		
	1	Getting started
	2	SMART^a^ goals
	3	Barriers
	4	Enlisting support (who and how)
**Month 2**		
	5	Accountability
	6	Making exercise a priority
	7	Having fun
	8	Overcoming roadblocks
**Month 3**		
	9	Benefits of exercise
	10	Staying motivated
	11	Revisiting goals: are your goals realistic?
	12	Managing stress
**Month 4**		
	13	Problem solving
	14	Advocating for yourself
	15	Enhancing support networks
	16	Planning for exercise maintenance

^a^SMART: Specific, Measurable, Achievable, Realistic, Timely.

### Content Changes

The main content changes that participants encouraged were reducing the volume of text and using images and videos to convey that content. After the redesign, all 16 e-modules included a short video segment (eg, 1 to 3 min) that featured various people who experienced spinal cord dysfunction and who are vocal advocates or disability researchers. Each video addressed a specific aspect of the week’s topic, such as ideas for helping to make or keep exercise fun. Bulleted text appeared beside the videos to highlight the main points the speaker addressed during the segment. Each person featured in a video gave a brief video introduction of themselves and were placed on the Peer Mentor tab. Changes implemented were guided by the Agency for Healthcare Research and Quality’s health literacy toolkit [32] to ensure usability for consumers from varying backgrounds (eg, levels of education). Site changes used simplified language, large text, and more images to facilitate access and understanding for the average user.

**Figure 2:**
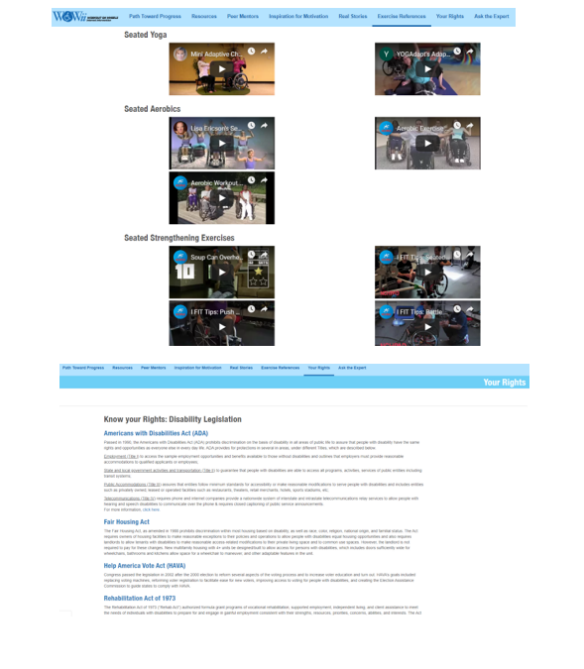
Resource pages.

The participants also recommended compiling additional resources that could be displayed on the website and to where participants could refer if they had questions about equipment, facilities, or their rights as a person with a disability. The array of ideas participants generated included adding resources to facilitate participants’ access to activities and programs to support exercise adoption. To address this, the team compiled a list of local resources for physical activity that included accessible gym and recreation facilities and local durable medical equipment suppliers; added embedded links to accessible exercise videos (eg, seated yoga and wheelchair dance; see Figure 2); provided informational resources that included descriptions of disability-specific legislation (eg, Americans with Disabilities Act and Fair Housing Act); and listed contact information for governmental and social resources (eg, independent living centers and disability magazine). These were added via tabs that run across the top of the Web page.

### Program Delivery and Functionality Changes

**Figure 3:**
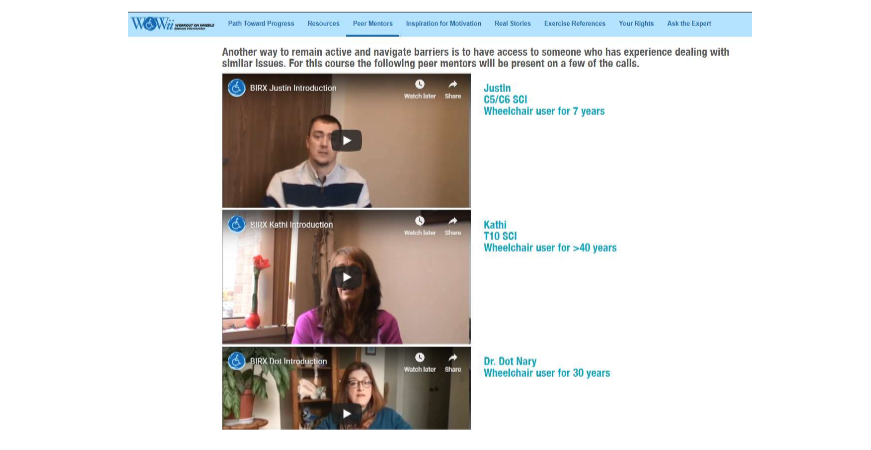
Mentor page.

The main change related to program delivery was to include people with SCI as part of the team helping guide participants through WOWii as they begin and follow through with their exercise program. On the basis of this input, the study team invited 2 people (1 male and 1 female) with SCI who are both regularly physically active to formally serve in the role of *peer mentors* and be paid an annual stipend to serve in this consultant role (Figure 3). The mentors have several explicit roles in the project which include helping to colead several of the virtual weekly modules, facilitate online group discussions during these sessions, be available to answer questions submitted to the website’s WOWii *Ask the Expert* button, and provide support by phone to WOWii participants who may be struggling with their exercise program.

A total of 3 new features to enhance functionality of the WOWii site were added to address participants’ ideas for helping users stay on track with their new exercise program. They encouraged adding (1) the ability for participants to receive text reminders for each workout, based on their individual workout schedules that users could opt *in* or *out* of to receive. They also proposed including (2) an interactive calendar feature that would allow them to view their weekly exercise progress in relation to their weekly exercise goals. Another idea that was suggested was creating (3) a tab that would display a rotating view of motivational pictures and quotes that encourage participants to stick with their exercise program. In addition, the participants discussed the potential benefits of including a calendar to show upcoming events and an individualized energy expenditure guide. Neither suggestion was implemented owing to the time required to create and maintain these tools regularly. The energy expenditure guide would also have presented exorbitant financial costs as the values are not easily available.

## Discussion

### Principal Findings

Using a PAR approach to formally include men and women with SCI who represented different impairment levels and exercise histories to collaborate with our team in revamping the WOWii website led to a substantial redesign of the site. The revamped site had less text, greater functionality, and more images of people with SCI throughout. The iterative process allowed our SCI partners to provide input as to whether their ideas regarding the organization, content, and functionality were accurately implemented on the redesigned site (eg, intuitive navigation, inclusion of video, reduced text, and easy access to media assets). The redesigned WOWii site contains all the material provided to participants during the educational workshop yet presented in a manner that PAR participants reported was easy to access, follow, and understand (see Multimedia Appendix 2 for the graphic that depicts changes implemented from the original into the redesigned site). The online information addresses SCI-specific exercise benefits, presents types of activities that people with various levels of SCI can perform, shows equipment, and provides links to available resources, as well as delivers step-by-step instructions about how to safely begin and continue an exercise program.

Although few published studies have investigated using Web-based platforms to improve health, SCI-specific health resources are available online, including sites that address physical activity. In their review of 30 SCI-focused websites, Jetha et al [33] reported that SCI-specific physical activity information is available, but the sites mostly present general information about the benefits of and barriers to physical activity. The authors note that few sites provide theoretically based intervention strategies to help people with SCI become more physically active and state that there is a need to improve information available on the internet about physical activity for people with SCI to enhance their access to quality information including interactive opportunities for developing behavioral skills. The collaborative relationship among those with SCI who provided iterative rounds of feedback on the WOWii site led to developing an online resource that addresses several of these shortcomings, in particular related to including theoretically based approaches to promote physical activity and adding interactive opportunities to develop these behavioral skills. Additional study is warranted to examine whether this Web resource is a feasible and effective platform for increasing physical activity among those with SCI.

Notably, a resource similar to the WOWii program is offered by the National Center on Health, Physical Activity, and Disability, which is known as 14 Weeks to a Healthier You. Both Web-based programs offer similar content in terms of informational resources and content regarding aerobic and strength training, and allow participants to schedule their workouts, track their exercise and diet, and opt in for text reminders. However, the programs differ in terms of delivery and participant interaction. The Healthier You program guides people through starting and keeping up with a physical activity program by delivering weekly emails with links to physical activity videos and content on the Healthier You website that participants move through at their own pace, whereas the WOWii program guides participants through the 16 weeks by hosting weekly 60-min group-based virtual sessions where a group of 10 to 14 participants meet over a virtual platform (Zoom) facilitated by a WOWii staff member who introduces the skills-based topic addressed in that week’s module and facilitates discussion among and sharing by the group members. These weekly virtual sessions are designed to facilitate group cohesion and have members serve as a support network and accountability, allowing enrollees to connect with other program users by adding them as a friend on the Healthier You site.

### Limitations

Feedback for adapting the website came from a small convenience sample of individuals with SCI who were not representative of the broader population living with SCI. The sample had more education, higher employment, and was less racially diverse than observed among the broader SCI population. Thus, including individuals with more diverse racial, educational, and employment backgrounds may have allowed those with different internet experiences to have potentially provided other recommendations for changing the content and function of the website.

### Conclusions

Using an iterative PAR approach to collaborate with individuals who represent the target audience for this intervention allowed for receiving substantial input and guidance that transformed the layout and functionality of the WOWii internet site. Participants shared their insights about what they would like to see and have available on the site. Feasibility of using the redesigned website by individuals with SCI will be tested in a 4-week trial, and effectiveness of the 16-week WOWii program will be investigated in a subsequent randomized controlled trial.

## Appendix

Multimedia Appendix 1 WOWii redesign.

Multimedia Appendix 2 Diagram of website changes.

## Abbreviations

ACL: Administration for Community Living
CMD: cardiometabolic disease
HHS: Health and Human Services
NIDILRR: National Institute on Disability, Independent Living, and Rehabilitation Research
PAR: Participatory action research
SCI: spinal cord injury
WOW: Workout on Wheels
WOWii: Workout on Wheels internet intervention
