# Supplementary figures and images for: Website Redesign of a 16-Week Exercise Intervention for People With Spinal Cord Injury by Using Participatory Action Research
Source: JMIR Rehabil Assist Technol. 2019 Dec 17;6(2):e13441. doi: 10.2196/13441 (PMC6938595; doi:10.2196/13441)

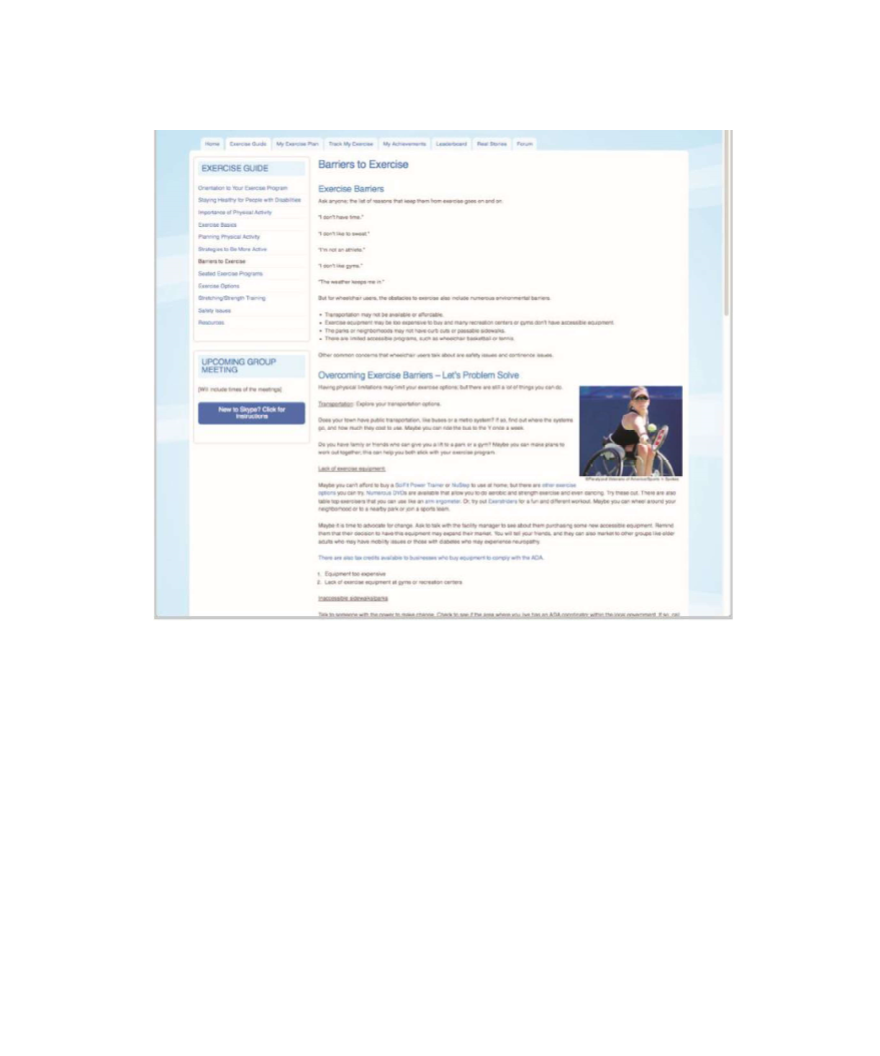

Supplement: Multimedia Appendix 1 [file rehab_v6i2e13441_app1.png]

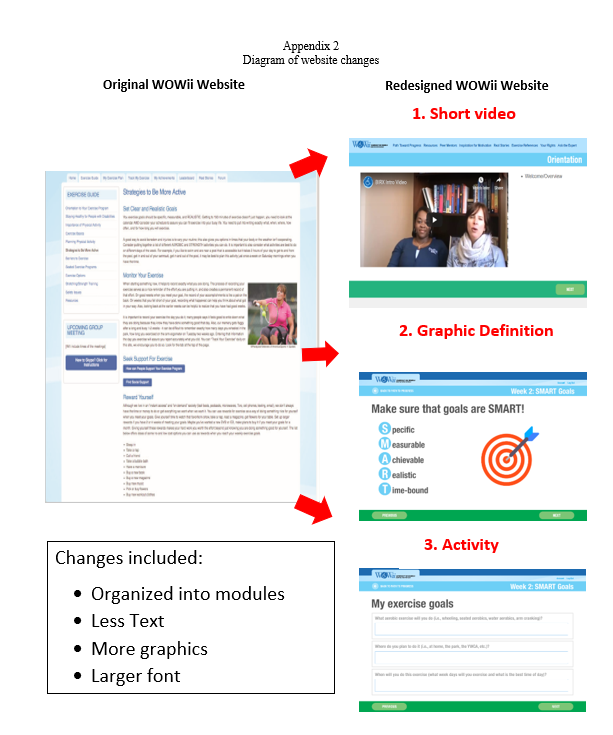

Supplement: Multimedia Appendix 2 [file rehab_v6i2e13441_app2.png]
